# Application of Wireless Power Transmission Systems in Wireless Capsule Endoscopy: An Overview

**DOI:** 10.3390/s140610929

**Published:** 2014-06-19

**Authors:** Md Rubel Basar, Mohd Yazed Ahmad, Jongman Cho, Fatimah Ibrahim

**Affiliations:** 1 Department of Biomedical Engineering, Faculty of Engineering, University of Malaya, Kuala Lumpur 50603, Malaysia; E-Mail: rubel24434@gmail.com; 2 Department of Biomedical Engineering, Inje University, Gimhae 621-749, Korea; E-Mail: minerva@inje.ac.kr; 3 Center of Innovation in Medical Engineering, Faculty of Engineering, University of Malaya, Kuala Lumpur 50603, Malaysia; E-Mail: fatimah@um.edu.my

**Keywords:** wireless power transmission (WPT), wireless capsule endoscopy (WCE), robotic capsule endoscopy (RCE), inductive coupling, specific absorption rate (SAR), current density

## Abstract

Wireless capsule endoscopy (WCE) is a promising technology for direct diagnosis of the entire small bowel to detect lethal diseases, including cancer and obscure gastrointestinal bleeding (OGIB). To improve the quality of diagnosis, some vital specifications of WCE such as image resolution, frame rate and working time need to be improved. Additionally, future multi-functioning robotic capsule endoscopy (RCE) units may utilize advanced features such as active system control over capsule motion, drug delivery systems, semi-surgical tools and biopsy. However, the inclusion of the above advanced features demands additional power that make conventional power source methods impractical. In this regards, wireless power transmission (WPT) system has received attention among researchers to overcome this problem. Systematic reviews on techniques of using WPT for WCE are limited, especially when involving the recent technological advancements. This paper aims to fill that gap by providing a systematic review with emphasis on the aspects related to the amount of transmitted power, the power transmission efficiency, the system stability and patient safety. It is noted that, thus far the development of WPT system for this WCE application is still in initial stage and there is room for improvements, especially involving system efficiency, stability, and the patient safety aspects.

## 1. Introduction

The gastrointestinal (GI) or digestive system extracts the essence of our diet and keeps our body active, but nowadays an increasing number of people are being affected by GI disorders [1,2] due to malignant diseases like gastric cancer [3,4], tumors [5,6] and bleeding [7,8] to name a few. Early detection is important for effective prevention and treatment that can prevent subsequent complications or diseases. Advanced endoscopy has a great potential to serve this purpose of early detection screening. It can provide comfortable, painless diagnosis to the patient and may reduce the time span for the patient to stay in a health care facility and more importantly, it makes it possible for a thorough diagnosis to be performed seamlessly without interrupting the patient's daily activities. This is possible because wireless capsule endoscopes (WCEs) are able to travel to the parts where the typical endoscopes fails to reach such as small bowel area [9–11], and this can be done without full supervision by a specialist.

For over a decade, since the invention of WCE [12,13] it is the only technique that can allow direct visualization of the full length of the GI tract including the small bowel for many pathologies [9,14,15]. Thus far, several clinical products have been developed [16–18] with different specifications and for use in different parts of the GI tract. Nonetheless, the diagnostic yield (DY) [11,19,20] remains the main issue because WCE is still considered an immature technology and there are many features that require improvement. To obtain a better diagnostic yield, important features such as image resolution, frame rate, working time, and view angle need to be improved [16,21]. Further, additional features such as miniaturized active control systems, capsules' actuation and locomotion mechanism, can provide a better generation of WCE devices whereby a robotic type of capsule endoscopy (RCE) can be possible [21].

In RCE, the magnetic based actuation mechanism is one of the efficient control methods. This method consists of a small permanent magnet embedded inside the capsule which interacts with the magnetic field generated by a large external magnetic source resulting in an actuation force [22,23]. A similar principle also can be used for localization of the capsule as reported in [24]. The main advantage of such magnetic-based control is that it minimizes power demand at the RCE, because the power required at the RCE to achieve actuation and locomotion are almost negligible. Due to this capability, research in this field is still ongoing [25–27]. However, such magnetic-based RCE systems possess limitations in terms of portability, unlike other types of RCE systems such as on-board micro motor [28] and shape memory alloy (SMA) [29]-based portable RCEs. These kinds of portable active actuation and locomotion RCE systems, however, require high peak power [30]. The VECTOR Project (supported by the European Union) revealed that the peak power required will be at least few hundred mW after taking into consideration all the on board power-consuming modules within a RCE [30,31]. Moreover, some advanced features including microsurgery, drug delivery and biopsy are also anticipated for multifunctional medical robot (MMR) WCE [32,33] that will also increase the power demands of the new generation of WCE systems. Accordingly, it can be concluded that the peak power requirement for the new generation of WCE is becoming much higher than that of the existing WCE units and the battery capacity may not be sufficient [30,34,35]. This is the main bottleneck in realizing the new generation of WCE devices [36,37].

Considering the compact dimensions and recent battery technologies used in the existing WCE, the amount of power deliverable by a typical WCE battery is merely around 25 mW [37,38]. On the other hand, although the harvesting of energy from the ambient sources such as light, radio frequency, thermo-electric, or vibrations is able to alternatively generate electrical energy, however the power output is still relatively low and in the range of μW [39,40]. In this regard, the technique of wireless power transmission (WPT) that employs a transmitting coil (TC) positioned outside the human body with a receiving coil (RC) installed within the WCE might be the best candidate to overcome the above limitations as this technology is able to provide relatively higher levels of power to WCE. Further, it also offers flexibility for power adjustment that allows the transmission of the right amount of power.

The WPT technology has been proven to be useful to power up healthcare devices, especially in biomedical implants [41–43]. However, unlike the other biomedical implantable devices such as retinal implants, neural recorders and pacemakers, the WPT approach in WCE faces additional challenges due to: (i) the significant distance between the transmitting and receiving coil; (ii) unpredictable capsule orientation and motion; and (iii) the need for compact size of the RC and its power harvesting circuits [35,44–46]. To overcome these challenges, the WPT system targeted for WCE must be carefully designed and optimized so that it can satisfy the desired design requirements and its operation must not cause interference or adverse health effects to the patient. Among the important aspects that need special investigation are the delivered power that must be sufficient and stable, irrespective of capsule movement. In addition, the dimensions of the receiving antenna and its power harvesting circuit must be compact enough to be assembled in the capsule. Moreover, the safety verification on the operation of the WPT systems for WCE is indispensable where special consideration must be given on several safety indexes such as: specific absorption rate (SAR); current density induced in human tissues; and overheating of the WPT RC. These parameters must not exceed the known safety limits [47–49].

There are studies that have been reported in the open literature describing the development of a WPT system for WCE. However, systematic reviews considering recent technological developments of WPT employed for future WCE are limited. Thus, this paper presents a comprehensive review of the recent studies on the development of emerging WPT systems for future WCE with major focus on the following aspects: (i) the amount of received power by the WCE; (ii) link efficiency between the transmitter and the receiver; (iii) safety and compatibility of WPT in WCE; (iv) transmitter-receiver link stability and the causes of its instability. In addition, the critical observations and recommendations for future research for next generation of improved WCE are also discussed.

## 2. Power Requirement for Future WCE

**Figure 1.**
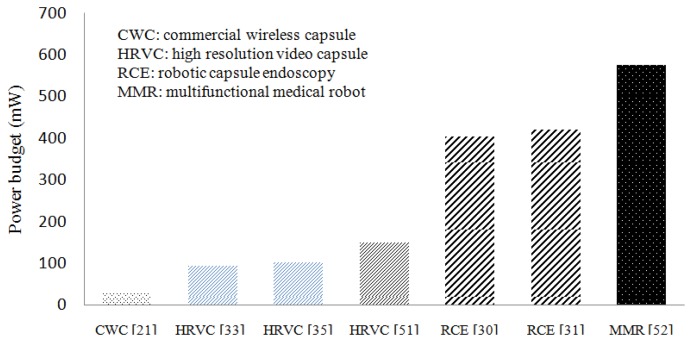
Power requirement for different types of WCE instruments.

The power requirement for WCE increases with the increase of the quality of the captured image and the addition of the advanced features as illustrated in Figure 1. The most basic commercial wireless capsule (CWC) typically requires around 30 mW of electrical power [21,50], mainly to power up a CMOS image sensor, light emitting diodes (LEDs), and RF transceiver. One of the main limitations of CWC is that the image quality is suboptimal, which may hinder further diagnosis from the recorded images. This can be overcome by employing high resolution video capsules (HRVCs) [33,35,51] which utilize higher resolution imaging/video devices, higher light intensity, and higher RF transmitter bandwidth for higher quality image data transmission. All these features, however, increase the power requirement for HRVCs that ranges up to 200 mW as illustrated in Figure 1. The next generation of WCE known as robotic capsules (RCEs) will have additional sub-systems such as advanced control electronics, locomotion systems, auto focus systems, image compression and other sensors [30,31]. Such RCEs demand a higher level of electrical energy that can reach up to 400 mW as illustrated in Figure 1. The inclusion of more actuators for multi-functional feature WCE (MMR) may further increase the needs for higher electrical power that can reach around 570 mW [52]. Based on these anticipated features, the estimated power requirement for the next generation capsules is far beyond the capacity of existing advanced battery technologies, therefore, an alternative approach is required. In this regards, the vision of WPT systems is to meet this increasing power demand for the next generation WCE with higher system efficiency, stability, safety and portability.

## 3. WPT System for WCE

**Figure 2.**
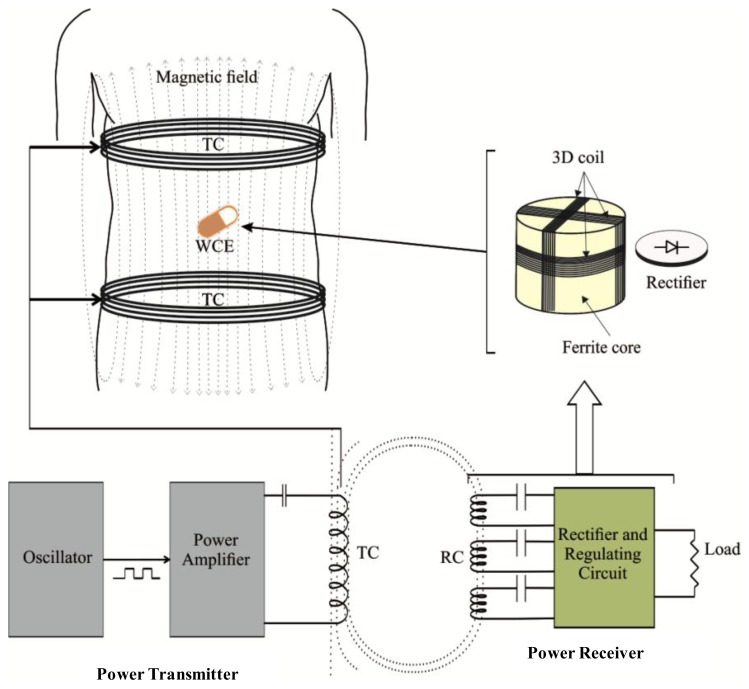
Illustration of WPT system in WCE environment.

Wireless power transmission (WPT) system refers to a system that transfers electrical power wirelessly from a transmitter to a receiver in the form of electromagnetic waves. WPT systems can be divided into two different categories [53,54]: (i) radiative coupling; and (ii) inductive coupling. The best choice of any particular method is highly dependent on its application area. The radiative technique is well suited for applications that require far field transmission that generally involves farther separation between the transmitter and the receiver, whereas the inductive coupling is more suitable for near field applications where the distance is relatively shorter [55]. Considering the application environments, near field inductive coupling, based on electromagnetic induction between two coils, is well accepted for medical applications, and this includes wireless capsule endoscopy (WCE) [56,57]. A WPT system based on induction coupling mainly consists of two subsystems, the power transmitter and the power receiver, as illustrated in Figure 2. Generally, the power transmission section comprises an oscillator, a power amplifier circuit and a transmitting coil (TC) transverse to the body. On the other hand, the receiving section comprises of a three receiving coil (RC) set wound around a ferrite core, a rectifier and a regulating circuit. All the sub-components of the receiver are compacted inside the capsule. Both the TC and the RC resonate at the same frequency. A series resonating circuit (SRC) or parallel resonating circuit (PRC) is typically employed at the TC and RC to obtain resonance. The oscillator generates the desired excitation signal which is then amplified by the power amplifier. The power amplifier makes it possible for high amplitude electrical current to be supplied to the TC so that it produces a strong alternating magnetic field. This magnetic field interacts with the RC and induces an alternating current in the RC. It then is turned into a direct current (DC) by the rectifier, and finally the DC is smoothened and stabilized by the regulating circuit. For practical use of WPT system, it must fulfill the following requirements: (i) it must be able to deliver stable and sufficient power to ensure the smooth working of the WCE; (ii) the level of the electromagnetic field generated from TC must not harmful to human tissues and organs; and finally (iii) the receiving coil (RC) and its regulation circuit should be small enough so that it can be assembled into a small size capsule. The aforementioned conditions imposed challenges in WPT system design targeted for WCE.

### 3.1. Inductive Power Transmitter

**Figure 3.**
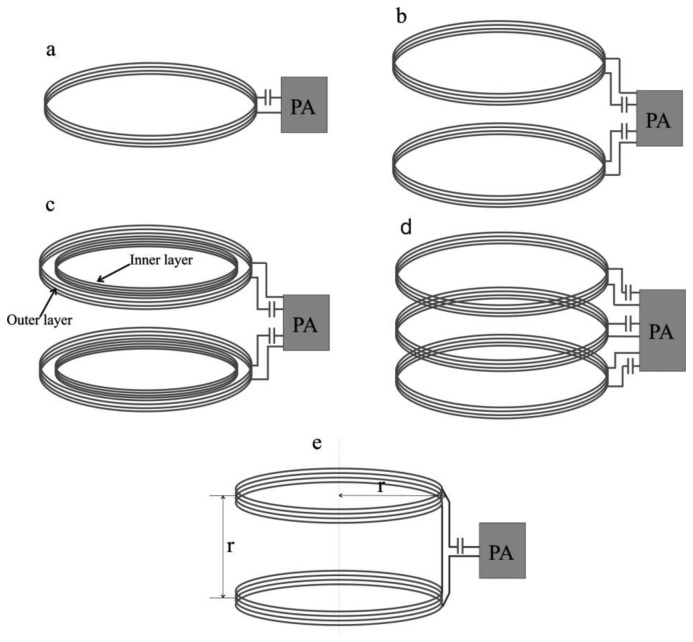
Common structures of TC: (**a**) solenoid; (**b**) pair of solenoid; (**c**) pair of double layer solenoid; (**d**) segmented solenoid; (**e**) Helmholtz coil. PA = power amplifier.

The inductive power transmitter includes a power amplifier (PA) and transmitting coil (TC). The power amplifier amplifies the TC driving signal that is usually in the form of a square wave [36,58] (with 50% duty cycle) or a sinusoidal wave [59]. The transmitting coil induces magnetic fields when the driving signal is applied to it and the intensity of the induced magnetic field depends on the magnitude of the TC driving signal. In the power amplifier, the typical mode of amplifications includes a class D or class E amplifier. Although both the class D and class E amplifiers are able to amplify the driving signal, class E is widely used since it has a better efficiency (theoretically it can achieve 100% efficiency) [60,61]. However, the performance of class E amplifiers is highly sensitive to the parameters of the load network (inductance of TC and resonating capacitor). Due to manufacturing errors in the resonating capacitor and the environmental effects on the TC inductance, it is difficult to maintain load impedance absolutely which has led to the utilization of class D amplifiers in some studies [62,63]. Although, the intensity of the generated magnetic field depends on the magnitude of the TC driving signal, the uniformity of the field depends completely on the type and the design structure of the TC [64]. In general, they are two types of TC which include solenoid coils [12,36,61–63,65–69] and Helmholtz coils [30,33,70]. The solenoid coil can be arranged in four different forms: (i) single solenoid [65]; (ii) a pair of solenoids [67]; (iii) a pair of double layer solenoids [12] and (iv) segmented solenoids [63]. The generalized structures of these coils are shown in Figure 3a–e.

Each of the structures has their own advantages. Owing to its simple structure, a solenoid coil is easy to design and implement, but its main drawback is non-uniformity of the induced magnetic field within its inner region as a result of the fact that the received power at the receiver may not always be stable [51,62]. In addition, this non-uniformity increases the risk to the tissues present near the transmitting coil, especially when the supplied current to the coil has to be increased. A better uniformity of the magnetic field within the inner region can be achieved by using Helmholtz coils [30,62]. This way, the Helmholtz coil configuration allows good confinement of the magnetic field in the patient's body, and hence may reduce the risk of unnecessary exposure.

A comparative study by [68] suggests that the field uniformity is the best in Helmholtz coils, moderate in a pair of solenoid coils and less uniform in a single solenoid coil. Due to non-uniformity, the magnetic flux density induced by solenoid and a pair of solenoid coils can achieve up to 200 μT higher than the density in Helmholtz coils under similar excitation. For any coil type, its diameter, number of turns and wire gauge are the main important design parameters which determine the quality of TC. The selection of these parameters in existing studies has been summarized in Table 1. The diameter of TC is one of the vital parameters which has been chosen in a wide range from 30 to 75 cm. Although, the smallest TC diameter of 30 cm may not be compatible for healthy and obese patients, nonetheless it has been used in many studies to validate their prototype of WPT systems [63,65]. In general, a smaller diameter of TC improves efficiency because it allows a better coupling between TC and RC. However, it may increase the electromagnetic exposure on the patient's body because the close proximity of the TC winding to the user. Use of larger TC can overcome this problem but a higher power will be required for driving the coil.

### 3.2. Inductive Power Receiver

The receiving subsystem consists of a power receiver coil (RC) wound around a ferrite core with a rectifier and a regulator circuit. In principle, the presence of a time varying magnetic field perpendicular to the cross sectional area of the receiver coil induces an AC current in it and as a result an AC potential is generated at the terminals of the receiver coil. The rectifier circuit is used to rectify the AC source into a DC source, and the regulator circuit stabilizes the DC level so that it is usable to power up the WCE circuitries. Factors affecting the received power include: (i) the RC dimensions; (ii) the relative alignment between TC and RC; (iii) the ferrite core used; (iv) the matching between RC and load impedance; (v) the quality factor of TC and RC; and (vi) rectifier circuit after the RC.

#### 3.2.1. RC Dimensions

The dimensions of the power receiving coil (RC) determine the induced voltage at the RC. A larger cross sectional area would result a higher voltage. However, small bowel capsules that are typically used have merely about 11 mm in diameter and about 26 mm in length [16]. These impose size limitations on the RC. In order to simplify integration of the receiving subsystem without the need of significant changes in existing capsule architectures, the diameter of the RC (d_RC_) must be smaller than the diameter of the existing capsules (*i.e.*, d_RC_ < 11 mm). The simplest option is to choose the diameter to be about the diameter of the battery typically employed in WCE [69], that is around 8 mm. The RC diameters used in existing research are shown in Table 2. The induced voltage at the receiver also relies on the maximum possible number of turns within the nominated dimensions. Usually the 3D RC uses three coils with an equal number of turns. To get the maximum number of turns, a lower diameter of (high wire gauge AWG 44/40/33) enameled wire is typically used.

#### 3.2.2. Alignment between TC and RC

Another factor affecting the received energy is the orientation of the RC with respect to the TC. Ideally, the cross-sectional area of the coil must always be perpendicular to the direction of the magnetic field emanated from the transmitting coil to ensure maximum and stable power reception. However, this cannot always be guaranteed because the capsule carrying the receiving coil has to pass through the digestive system which has an irregular path. Therefore, there can be instances where the RC and TC become misaligned, thus reducing the received DC power. Three-dimensional RC where three coils are set perpendicular to each other can be employed to minimize this problem, but, as the three coils are wound on each another, the diameter of each coil is not identical hence the self-inductance and the effective series resistance (ESR) of the three coils become dissimilar as indicated in Table 2. These contribute further to the fluctuations of the received power, especially with changes of the alignment of RC with respect to TC.

#### 3.2.3. Ferrite Cores Used

Utilization of a ferrite core with high initial permeability (μi) can increase the magnetic field intensity and hence boost the received power of the RC [31]. The commonly used ferrite cores are 3F4 of Ferroxcube (μi = 1000) [71], R5K (μi = 5000) [62] or R10K (μi = 10,000) [12] of DMEGC Magnetic Co., Ltd., Zhejiang, China.

#### 3.2.4. Matching RC with Load Resistance

The effective series resistance (ESR) is determined by the number of turns and wire gauge. A study by [65] revealed that, for a series resonance circuit (SRC), matching the ESR with the load resistance results in maximum power transfer from RC to load.

#### 3.2.5. Quality Factor

Quality factor (Q) is another important parameter which refers to the ratio of stored energy and energy losses by the coil at the certain frequency. The optimum Q value results in a higher system efficiency. At a certain frequency, Q value increases with the decreasing wire gauge and number of turns [62].

#### 3.2.6. Rectifier Circuit

Either full-bridge or full-wave schemes can be used as a rectifier circuit. Although both of these schemes have the same efficiency, the full-wave scheme requires fewer diodes than the full-bridge one, thus the use of the full-wave rectifier can save components and the space for the receiving circuit. On the other hand, a full-wave scheme requires a double number of turns for the same received power, which increases the RC size. The p-n junction diodes used in rectifier circuits typically have a forward voltage drop of around 350 mV at 100 mA current, and it has been reported in [62] that the full-bridge employing such a diode causes a power loss of around 75 mW. In order to overcome this problem, a switch mode efficiency enhancement rectifier circuit has been implemented in [73] using CMOS technology (UMC 0.18 μm) and the rectifying efficiency was boosted up to 93.6% which is 13.4% better than earlier designs.

## 4. Safety Limitations for Biological Tissues

The human body has complex electrical properties as its permittivity and conductivity change depending on the frequency and type of tissue [74]. The conducting properties of body tissue not only interfere with power transmission, but also give rise to the patient's body safety issue [75]. Excessive electromagnetic exposure may cause unexpected harm to human organs and their functionality. Thus, the patient's tissue safety cannot be ignored in the application of WPT. Electromagnetic waves can cause two kinds of influences: thermal effects and stimulant action [62] where the specific absorption rate (SAR) and current density are often used to indicate these two influences, respectively. The WPT system is safe for the patient's body if the SAR and the current density do not exceed the limits specified by the relevant regulations such as the International Commission on Non-Ionizing Radiation Protection (ICNIRP) [47,49]. Excessive received power may cause overheating of the RC, but according to the Japan Society of Medical Electronics and Biological Engineering (JSMEBE) the temperature below 42.5 °C is safe for the tissues surrounding the RC [48]. The safety specifications for WPT systems, standardized by ICNIRP and JSMEBE are given in Table 3.

## 5. Overview of Existing WPT Systems

### 5.1. Existing WPT System Performance

The existing studies on wireless power transmission (WPT) systems for wireless capsule endoscopy (WCE) have been selected from our comprehensive literature search. They are reviewed carefully by focusing on the amount of transmitted power and the overall transmission efficiency. Design parameters are analyzed and discussed in two categories namely: (I) Solenoid coil-based WPT systems and (II) Helmholtz coil-based WPT systems.

#### 5.1.1. Solenoid Coil-Based WPT Systems

The solenoid coil based WPT systems are summarized in Table 4. The simplest WPT system design using a single solenoid coil was proposed by Lenaerts *et al.* [61] and by Guanying *et al.* [65]. The efficiency of these systems was very low as a consequence of the lower amount of transmitted power due to safety considerations. In order to maintain the electromagnetic safety of the patient's body, Lenaerts *et al.* used electrical shielding on the TC, though this technique improves the safety level but degrades the efficiency. Guanying *et al.* compared the advantages and shortcomings of using a series resonating circuit (SRC) and parallel resonating circuit (PRC) on the transmitting and receiving side and revealed that SRC is better for the transmitting coil TC and for the receiving coil (RC) when the load resistance is low. A new approach load-adaptive power converter circuit with RC and end-fire helix emitter at TC introduced by Sun *et al.* in [46] improves the WPT efficiency. Additionally, Sun *et al.* also developed a high efficiency (93.6%) switch-mode rectifier and obtained whole WPT system efficiency of up to 3.04% [73]. In addition, Huang *et al.* [63] proposed that using a segmented TC, a group of solenoid coils arranged vertically can improve the efficiency of WPT systems. This kind of segmented TC improves the magnetic field intensity inside the TC that helps to increase the transmission efficiency by up to 3.8%.

Moreover, a pair of double layer solenoids as reported by Jia *et al.* [36] further increases the magnetic field sufficiently to obtain overall efficiency of up to 5.04%. However, after optimization of this double layer solenoid coil based WPT system in terms of size and safety consideration, the efficiency was reduced to 4.08% [76].

#### 5.1.2. Helmholtz Coil-Based WPT Systems

The performance of the Helmholtz coil-based WPT systems is given in the Table 5. Li *et al.* conducted an initial study on WPT systems for powering an endoscopic robot with three sets of Helmholtz coils having a maximum diameter of 32 cm [70]. The coil sets were energized with 35 W input power at 50 kHz and the maximum received power obtained at the receiving coil is 490 mW with an overall efficiency of 1.4%. According to their investigation, the overall efficiency was so low because of the quality of the enamel wire (diameter of 1.4 mm) used in the Helmholtz coil. However, the large diameter of the TC is important to maintain a sufficient gap between the TC and the patient's body and to have more uniform magnetic field inside the TC circumference. The large diameter of the TC increases the relative distance between the TC and RC (considering the RC is located at the center of the TC) and as a result the transmitted power gets reduced. A large scale Helmholtz coil was developed by Ryu *et al.* in [69], Carta *et al.* in [31], Pan *et al.* in [33], and Xin *et al.* in [62]. The miniaturization of RCs with the advantage of a ferrite core was studied by Carta *et al*. The study found that a ferrite core can increase the received power by 120% as compared with the same size of air core RC, and at the same received power, a ferrite core allows a 50% reduction of RC size. The proposed WPT system was able to deliver 300 mW power to the RC with ferrite core. Nonetheless, the advantage of ferrite cores depends on their initial permeability (μi). The ferrite core of high initial permeability (μi = 5000) used by Xin *et al.* obtained a transmitted power of 310 mW. The above mentioned WPT systems were tested with an air medium between the TC and RC. When Rya *et al.* tested the performance of the WPT system in an animal body and from the observation of LED intensity they concluded that the biological tissue does not affect the transmitted power at 125 kHz frequency, but Pan *et al.* reported that when they covered the receiving unit with pig fat of 6 cm thickness the transmitted power was reduced by 8% due to the absorption of fat at 181 kHz frequency.

### 5.2. Stability of WPT Systems

**Figure 4.**
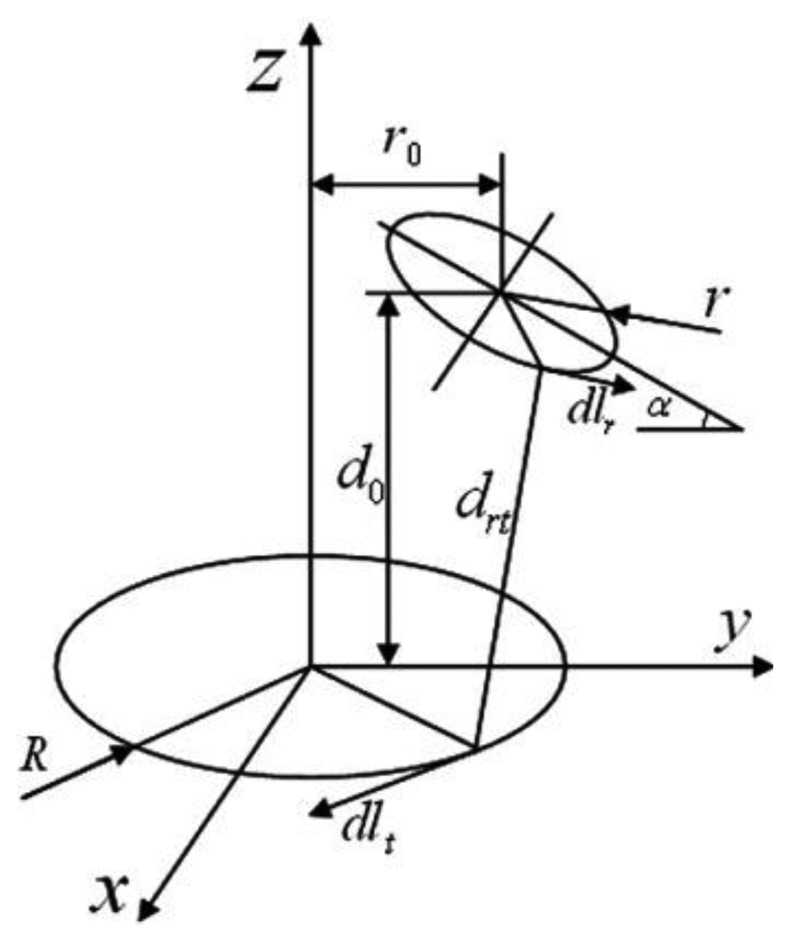
Relative alignment between TC and RC (adopted from [33]).

The stability of wireless power transmission (WPT) systems depends highly on the alignment between transmitting coil (TC) and receiving coil (RC). For the application in the wireless capsule endoscopy (WCE), the TC is fixed to the patient's body, but the RC has freedom of motion which may cause misalignment between the RC and TC, and as a result the received power at the load will vary. Referring to Figure 4, there are three kinds of RC misalignment: (i) axial misalignment (d_0_); (ii) lateral misalignment (r_0_); and (iii) pitch misalignment (α) as indicated in [33]. The effect of misalignment between RC and TC coils in WPT system can also be seen in different parameters such as coupling coefficient, efficiency, and received power of WPT system. The effect of misalignment observed in different studies is summarized in Table 6.

The stability, in terms of different parameters, is calculated based on the formula given in Equation (1), where, “Max” and “Min” are the maximum and minimum values of the observed parameters within the 15 cm distance from the centre axis of the cylindrical volume enclosed by the TC, respectively. The 15 cm distance is chosen considering the practical movement of RC during the operation. The level of stability for d_0_ and r_0_ depends on the TC design while the stability due to α relies on the RC design. It can be seen from the Table 6 that in term of d_0_/r_0_, Helmholtz coil-based systems [33,70] have better stability (maximum 83% stability) in comparison to the solenoid-based systems [12,65] which possess a maximum 40% stability.

(1) $$\text{Stability}=\left(1-\frac{\mathrm{Max}-\mathrm{Min}}{\mathrm{Max}}\right)\times 100\%$$

**Figure 5.**
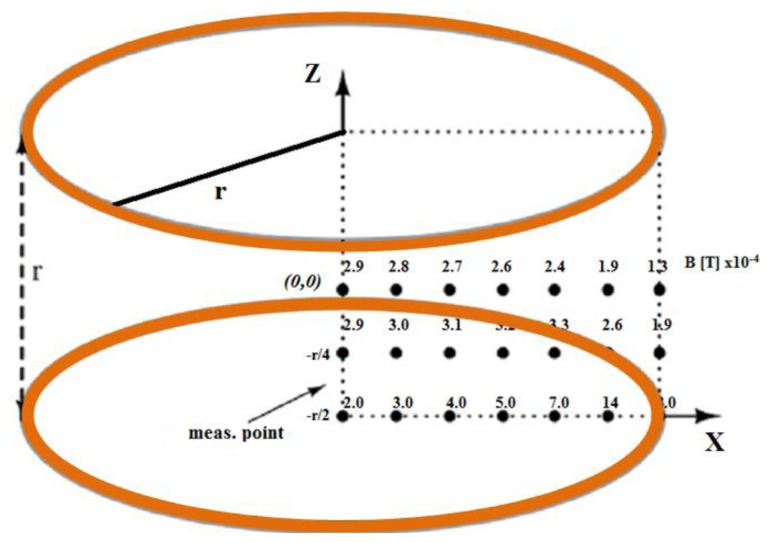
Magnetic field uniformity of Helmholtz coil (modified from [71]).

However, in terms of α, the stability of a Helmholtz coil-based WPT system proposed in [62] was poor (41%), mainly due to unequal electrical parameters of the RC set (inductance of coil 1: 478.5 μH; coil 2: 404.5 μH and coil 3: 390.9 μH). More accurate RC set design can improve the stability which occurs by the variation of α. For instance, if the RC sets have a closer inductance of each coil (coil 1: 97.1 μH; coil 2: 99.8 μH; coil 3: 97.1 μH) this results in 84% stability [30]. Looking beyond this, the adoption of a ferrite core is the great RC advancement but it had been pointed out in [65] that ferrite cores degrade the system stability more than air cores. Additionally, the three orthogonal pair TC can deliver more stable power than a single pair [70]. Moreover, although the ideal Helmholtz coil has a very uniform magnetic field inside it, this is difficult to achieve practically. The uniformity of the magnetic field in Helmholtz coil analyzed in [71] is shown in Figure 5. From the figure it is clear that there is significant non-uniformity.

### 5.3. Safety Verification of WPT Systems

**Figure 6.**
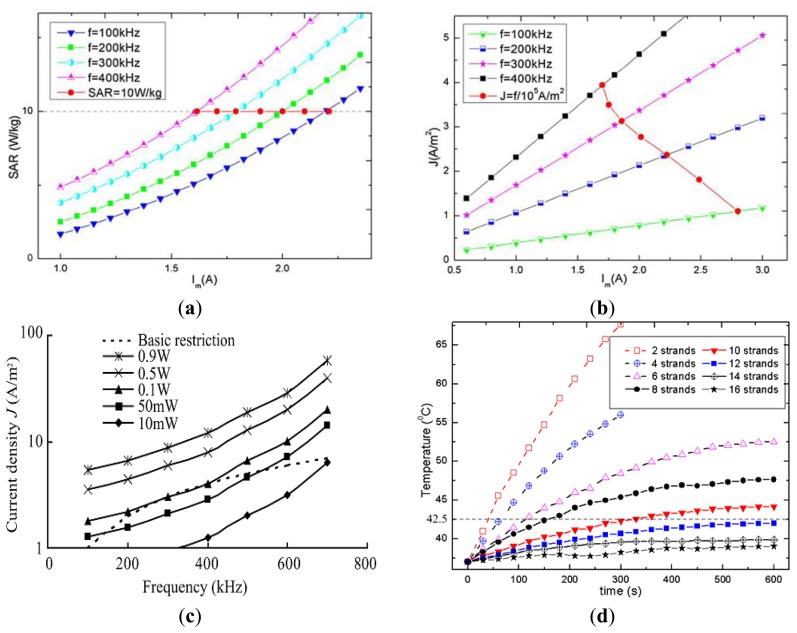
Variation of safety parameters with the design constants: (**a**) SAR with respect to frequency and TC current [12]; (**b**) Current density (J) with respect to frequency and TC current [12]; (**c**) Current density with respect to frequency and received power [75]; (**d**) temperature of RC with respect to wire strands [12].

The specific absorption rate (SAR) and current density (J) are induced in biological tissue due to exposure to the strong reactive magnetic field generated by the TC. These safety parameters depend on the frequency and the intensity of the field produced by the TC. In order to avoid potential health hazards, SAR and J should be below the levels suggested in the safety guidelines. However, direct measurement of SAR and J are very difficult and almost impractical, so often numerical simulations using human body models are utilized to predict these values. Analysis of SAR and J in WPT systems is shown in Table 7. Xin *et al.* [62] used a high resolution human body model with 56 different kinds of tissues to investigate the SAR and J. In the simulation model, the TC equivalent magnetic field generating coil was created and placed coaxially to the body model. Finally, the magnetic coil was excited at 400 kHz to generate the same amount of H field of the TC. By this model, the computed average SAR and J were found to be 0.392 W/kg and 3.82 A/m^2^ where the ICNIRP limits are 0.4 W/kg and 4 A/m^2^ respectively at 400 kHz. Jia *et al.* also used same kind of simulation scheme and observed the whole body SAR 8 W/kg and J 1.8 A/m^2^ in comparison to the limits of 10 W/kg and 2.18 A/m^2^ at 218 kHz. In addition, the SAR and/or J were found to be below the safety limit in [70,72]. The effective series resistance (ESR)-based SAR evaluation in [61] shown that in the patient's hands down condition, 7 A TC current at 1 MHz frequency results in 1.16 W/kg average SAR where the ICNIRP limit is 0.4 W/kg. Copper shielding of the inner and outer transmitting coil were used to control the excessive SAR, but this detunes the resonance frequency from 1 MHz to 1.4 MHz. Moreover, the SAR and J were analyzed by Shiba *et al.* [75] for the range of frequency from 100 kHz to 700 kHz and the results indicated that the J was less influential between 300 kHz to 400 kHz. In another study, Shiba *et al.* indicated that at 50 kHz, J greatly exceeded the restriction when the input current to the TC was 1.94 A. The SAR and current density are strongly related to the frequency and voltage/current applied to the TC, as highlighted in Figure 6a,b. The figures indicate the increasing trend of SAR and current density (J) when the excitation current and/or the excitation frequency are increased [12]. Also, it can be seen that SAR and current density may increase beyond the safety level with a higher frequency and/or TC current.

Despite that, transmission line modeling (TLM)-based simulation in [75] showed that it was possible to receive the maximum power of 100 mW (Figure 6c) while keeping the current density safe for a patient's body. There are few studies that include the investigation of RC temperature which mostly depends on the wire strands and core materials. Utilization of wire strands above 10 with ferrite core material resulted in an RC temperature below 42.5 °C [12,67]. The variation of RC temperature due to utilization of different wire strands studied in [76] is shown in Figure 6d.

## 6. Discussion

This paper has presented an overview on the development of emerging wireless power transmission (WPT) techniques for application in wireless capsule endoscopy (WCE). Based on the power budget compiled from several articles, it is noted that there is a large gap between the available battery power and the power required for the next generation of capsules. Depending on the capsule features, the required power may range from 93.4 mW (for a high resolution video capsule) [35] up to 570 mW (for a medical micro robot capsule) [52]. To cater to this high power requirement, various design approaches have been investigated. Most of the proposed methods optimized the design parameters associated with: structure and dimension of transmitting coil (TC) and receiving coil (RC); number of turns and utilization of wire strands for TC and RC; power amplifier; rectifier and resonance circuit; and resonance frequency. The best optimally designed WPT link so far is able to transfer about 540 mW DC power at the receiving coil [12]. Most of the existing studies merely tested the capability of the transferred power of their proposed WPT system in air medium (between TC and RC). However, the study in [33] suggests that the transferred power may be significantly reduced by the presence of biological tissue between TC and RC, thus, the *in vitro* test is necessary to validate the actual performance of the WPT link in the WCE environments. In addition, the self-directed movement and alignment of capsule in the GI path may cause misalignment between the RC and TC resulting in fluctuation of the received power at the RC, *i.e.*, the power at the receiver is not always stable. Two kinds of stabilities have been assessed in the literature, namely position stability (due to axial and lateral misalignment) and orientation stability (due to pitch misalignment). The optimum WPT system proposed in [62] obtained an orientation stability of 41.3% and a position stability of 82.1%. According to our stability comparisons in Table 5, the WPT link in [33] obtained 30% orientation stability and 63% position stability. The best orientation stability of 84% was obtained in [30]. Based on this work, it is noted that a uniform magnetic field increases the position stability; the orientation stability is related to the design of the RC-set. In particular, the use of a 3D RC that utilizes three coils wound around a ferrite core one on the other may have unequal electrical parameters due to the non-identical coils hence the received power may vary, even if they are experiencing equal amounts of magnetic field.

Frequency detuning is another important issue for the stability of WPT. Although, it is important to resonate the TC and RC at the same frequency, however in practice, the presence of body tissue or any other conducting object around the coil may detune the resonance frequency of the coils. The use of a copper sheet around the TC may further detune the resonance frequency of TC e.g., 1 MHz to 1.4 MHz as reported in [61]. In another case, the presence of body tissue detunes the resonance frequency of capsule antenna as indicated by [77,78]. Therefore, the consideration of frequency detuning is also important for a realistic WPT system.

Moreover, safety is a major consideration for the clinical use of WPT systems and it must be ensured. According to the safety analysis, the safety indexes “specific absorption rate” (SAR) and current density must always be within acceptable levels [12,62,75,79]. However, the current density may exceed the restrictions standardized by ICNIRP under certain system frequency and driving current levels applied to the TC. The current density increases along with increasing system frequency and driving current which impose the limit on the maximum system frequency and driving current to maintain the safety level [12,75]. The studies in [75,79] reveal that the SAR is mostly dependent on the terminal voltage of the transmitting coil (TC) but the current density varies according to the excitation current applied to the TC, so tuning of these two parameters may maintain the SAR and current density to be within the desired level. In addition, as the safety indexes are highly dependent on the voltage/current applied to the TC, the improvement of power transmission efficiency can make it easier to keep safety parameters well below the safety limit. As for the problem associated with RC temperature, this can be overcome by using multi-stranded wire as shown in [12].

## 7. Concluding Remarks

In this article, the existing studies on the wireless power transmission (WPT) for wireless capsule endoscopy (WCE) have been reviewed critically. The development of WPT in this area is still immature that there are many aspects that require improvements and further investigation to make the WPT system become practical in this area. Among the main problems we can highlight the non-uniformity of magnetic fields within the WCE operating region; the dissimilarity of electrical parameters at the receiver coils; and misalignment between receiver and the transmitter coils. All these contribute to fluctuation of the received power and affect system efficiency, therefore they must be further investigated to improve the feasibility of WPT. In addition, most of the existing WPT systems have been tested in air medium, however, *in vitro* tests are still necessary to fully validate the actual performance of WPT. The safety aspect of using WPT in WCE is another concern and the research on this aspect considering advanced WCE systems is still limited. We believe that the extensive research on WPT for WCE is necessary to realize advanced multi-functioning WCE devices.

## Figures and Tables

**Table 1.** Basic design parameters of transmitting coil used in existing studies.

Study	TC Type and Diameter (cm)	No. of Turns	Wire Gauge (AWG)	Self Inductance (μH)	DC Impedance (Ω)	Resonate at (kHz)
[65]	Solenoid Ø 30	25	16	368.7	NA	58.418
[33]	Helmholtz Ø 64	26	38	631	5	181
[62]	Helmholtz Ø 69	26	38	621	NA	400
[60]	Helmholtz Ø 75	12	12	147	1.5	1000
[52]	Solenoid Ø 40	66	16	347.6	0.3	36
[70]	Helmholtz Ø 32	25	15	NA	NA	50
[71]	Helmholtz Ø 30	16	12	187.5	NA	1000

**Table 2.** The design parameters of receiving coil.

Study	Coil	No of Turns	Self Inductance (μH)	ESR (Ω)	Diameter × Length (mm)	Wire Gauge (AWG)	Resonance Type	Frequency (kHz)
[33]	1	150	0.257	10	Ø 9.5 × 8.9	33	PRC	181
	2	150	0.090	11				
	3	150	0.235	12				
[31]	1	45	97.1	NA	Ø 9 × 7	40	Self	1000
	2	45	99.8	NA				
	3	45	97.1	NA				
[36]	1	160	286	6.6	Ø 13 × 13	44	SRC	218
	2	160	279	5.2				
	3	160	278	5.4				
[72]	1	33	43.2	NA	Ø 9 × 9	10	Self	1000
	2	33	43.7	NA				
	3	33	44.3	NA				
[62]	1	150	478.5	16	Ø 9.6 × 9.6	44	SRC	400
	2	140	404.5	13.3				
	3	130	390.9	13.5				

* SRC = series resonating circuit; PRC = parallel resonating circuit; Self = self-resonating.

**Table 3.** Basic safety limitation for human body tissue by ICNIRP and JSMEBE.

	By ICNIRP				By JSMEBE

Frequency Range	Localized SAR (W/kg)		Average SAR (W/kg)	Current Density (mA/m^2^)	Temperature of RC

	Head and Trunk	Limb			
1 kHz–100 kHz	—	—	—	<f (Hz)/100	<42.5 °C
100 kHz–10 MHz	<10	<20	<0.4	<f (Hz)/100	
10 MHZ–10 GHz	<10	<20	<0.4	—	

**Table 4.** Overview of solenoid coil-based WPT system performance.

Study	TC Size (cm)	RC Size (mm) Diameter, Length	Frequency (kHz)	Tx-Distance (cm)	Tx-Power (mW)	Link Efficiency
[61]	Ø 41	Ø 10, 13	1056	20.5	150	1%
Key observation: Posture of patent body has significant effect on the tuning of WPT system and the safety level, this effect can be reduced by using electrical shielding on the TC.						
Future work: Not suggested.						
[65]	Ø 30	Ø 10, 8	58.418	15	170	1.3%
Key observation: SRC performs well for higher load current but PRC for higher load voltage.						
Future work: In the future studies the improvement of coupling coefficient would be addressed.						
[46]	Ø 30	Ø 10, NA	24050	15	150	2.5%
Key observation: Two techniques End-Fire Helix Emitter at primary side and Load-Adaptive Power Converter at secondary side improve the power transmission efficiency.						
Future work: Not suggested						
[63]	Ø 30	Ø 11, NA	2000	15	NA	3.8%
Key observation: The segmentation of transmitting coil improves the transmission efficiency.						
Future work: The future study will be linked to the determination of optimum number of segmentation in transmitting coil.						
[73]	Ø 31	Ø 11, NA	1356	15.5	24	3.04%
Key observation: Switch-mode rectifier improves the rectification efficiency up to 93.6% which is 13.4% higher than the best previous designs.						
Future work: Not suggested.						
[12]	Ø 40	Ø 11.5, 11.5	218	20	540	5.05%
Key observation: Between SRC and PRC, SRC is more suitable for both of transmitting and receiving coil. Increasing either TC resistance or the intensity of magnetic flux can improve the system efficiency.						
Future work: The future research will focus on the development of a mathematical programming model to develop more practical and safe power transfer system.						
[76]	Ø 40	Ø 13, 13	218	20	500	4.08%
Key observation: Temperature in RC decreases if the RC is made with higher strands of enamel wire.						
Future work: In the future development WPT system, the size, safety and efficiency will be optimized.						

**Table 5.** Overview of Helmholtz coil-based system performance.

Study	TC Size (cm)	RC Size (mm) Diameter, Length	Frequency (kHz)	Tx-Distance (cm)	Rx-Power (mW)	Link Efficiency
[70]	Ø 32	Ø 10, 8	50	16	490	1.4%
Key observation: Three sets of Helmholtz coil improve the stability of WPT system but the large diameter of enamel wire used for Helmholtz coil result low efficiency.						
Future work: Not suggested.						
[69]	Ø 60	Ø 8, 5	125	5	300	N/A
Key observation: According to the observation of LED intensity, the performance of WPT system has not been affected by the animal body for choosing low frequency (125 **k**Hz) inductive link.						
Future work: Not suggested.						
[31]	Ø 60	Ø 9.5, 7	1000	30	300	N/A
Key observation: Ferrite core RC increases the received power by 120% than the same size of air core coil and ferrite core allowed to miniaturize the receiving coil-set by 52% keeping the same received power.						
Future work: Future study should be focused on full characterization of the source of external magnetic field.						
[33]	Ø 64	Ø 9.5, 8.9	181	32	136	N/A
Key observation: When the receiver was covered with a piece of pig fat (6 cm thick), the received power reduced about 8% (from 150 mW to 136 mW) because of the absorption loss by the biological tissue.						
Future work: Not suggested.						
[62]	Ø 64	Ø10, 12	400	32	310	1.24%
Key observation: The position stability of RC was better than the orientation stability as observed 82.1% and 41.3% respectively.						
Future work: In the future work, the *in vitro* experiment of WPT system with integrated locomotion system will be performed.						

**Table 6.** Overview of WPT system stability.

Study	TC Coil Type	Observed Parameter	Approximate Stability
[33]	Helmholtz	Coupling coefficient	63% (for d_0_) 83% (for r_0_)
[70]	Helmholtz	Efficiency	60% (for d_0_)
[12]	Double layer solenoid	Received power	40% (for d_0_)
[65]	Single solenoid	Coupling coefficient	32% (for d_0_, RC with core) 38% (for d_0_, RC without core)
[30]	Helmholtz	Received power	84% (for α)
[62]	Helmholtz	Received power	41% (for α)
[61]	Single solenoid	Efficiency	83% (for α)

**Table 7.** Overview of biological tissue safety study for various WPT systems.

Study	TC Type	Input Power	Frequency (kHz)	SAR (W/kg)	Current Density (A/m^2^)
[62]	Helmholtz	25 W	400	0.392 (av)	3.82
[12]	Solenoid	1.8 A	218	8 (wb)	1.8
[61]	Solenoid	7 A	1056	1.16 (av)	NA
[73]	power relay	8 W	1356	< 0.1(av)	NA
[70]	Helmholtz	35 W	50	NA	0.47

* av: average; wb: whole body.

## Notes

**Author Contributions** Two authors (Md Rubel Basar and Mohd Yazed Ahmad) extensively search the relevant studies from six electronics databases (Elsevier, PubMed, IEEEXplore digital library, SpringerLink, Google Scholar and Wiley Online Library), extract data from the selected studies and finally prepare this manuscript. Other authors, Fatimah Ibrahim and Jongman Cho, verify the critical analysis from the extracted data and guide for the organization of this paper.
